# Development and validation of a clinical risk model to predict the hospital mortality in ventilated patients with acute respiratory distress syndrome: a population-based study

**DOI:** 10.1186/s12890-022-02057-0

**Published:** 2022-07-11

**Authors:** Weiyan Ye, Rujian Li, Hanwen Liang, Yongbo Huang, Yonghao Xu, Yuchong Li, Limin Ou, Pu Mao, Xiaoqing Liu, Yimin Li

**Affiliations:** 1 Department of Critical Care Medicine, First Affiliated Hospital of Guangzhou Medical University, Guangzhou Medical University, Guangzhou, China; 2 State Key Laboratory of Respiratory Disease, National Clinical Research Center for Respiratory Disease, Guangzhou Institute of Respiratory Health, First Affiliated Hospital of Guangzhou Medical University, Guangzhou Medical University, Guangzhou, China; 3 The First Affiliated Hospital of Jinan University, Guangzhou, China

**Keywords:** Acute respiratory distress syndrome, Database, Mortality, Prediction, Ventilation

## Abstract

**Background:**

Large variability in mortality exists in patients of acute respiratory distress syndrome (ARDS), especially those with invasive ventilation. The aim of this study was to develop a model to predict risk of in-hospital death in ventilated ARDS patients.

**Methods:**

Ventilated patients with ARDS from two public databases (MIMIC-III and eICU-CRD) were randomly divided as training cohort and internal validation cohort. Least absolute shrinkage and selection operator (LASSO) and then Logistic regression was used to construct a predictive model with demographic, clinical, laboratory, comorbidities and ventilation variables ascertained at first 24 h of ICU admission and invasive ventilation. Our model was externally validated using data from another database (MIMIC-IV).

**Results:**

A total of 1075 adult patients from MIMIC-III and eICU were randomly divided into training cohort (70%, n = 752) and internal validation cohort (30%, n = 323). 521 patients were included from MIMIC-IV. From 176 potential predictors, 9 independent predictive factors were included in the final model. Five variables were ascertained within the first 24 h of ICU admission, including age (OR, 1.02; 95% CI: 1.01–1.03), mean of respiratory rate (OR, 1.04; 95% CI: 1.01–1.08), the maximum of INR (OR, 1.14; 95% CI: 1.03–1.31) and alveolo-arterial oxygen difference (OR, 1.002; 95% CI: 1.001–1.003) and the minimum of RDW (OR, 1.17; 95% CI: 1.09–1.27). And four variables were collected within the first 24 h of invasive ventilation: mean of temperature (OR, 0.70; 95% CI: 0.57–0.86), the maximum of lactate (OR, 1.15; 95% CI: 1.09–1.22), the minimum of blood urea nitrogen (OR, 1.02; 95% CI: 1.01–1.03) and white blood cell counts (OR, 1.03; 95% CI: 1.01–1.06). Our model achieved good discrimination (AUC: 0.77, 95% CI: 0.73–0.80) in training cohort but the performance declined in internal (AUC: 0.75, 95% CI: 0.69–0.80) and external validation cohort (0.70, 95% CI: 0.65–0.74) and showed modest calibration.

**Conclusions:**

A risk score based on routinely collected variables at the start of admission to ICU and invasive ventilation can predict mortality of ventilated ARDS patients, with a moderate performance.

**Supplementary Information:**

The online version contains supplementary material available at 10.1186/s12890-022-02057-0.

## Background

Acute respiratory distress syndrome (ARDS) is an acute diffuse inflammatory lung injury, featuring increased pulmonary vascular permeability and lung weight mechanistically, and clinically hypoxemia and bilateral radiographic opacities [1]. Although numerous interventions, for example, low tidal volume ventilation [2] and conservative fluid strategies [3], have been employed in the management of ARDS, whose hospital mortality remained significantly high in critical illness patients in intensive care unit (ICU), especially that of invasive ventilation [4]. In addition, the large variability in mortality exists in ARDS, which was thought to contribute to indeterminate or conflicting study results in most randomized clinical trials in patients with ARDS [5].

As risk stratification for ARDS would aid in medical decision making and clinical trial design, lots of effort have been made to develop a model of predicting ARDS-induced/related mortality [6–8]. Sequential Organ Failure Assessment (SOFA) [9] was initially designed for assessing organ dysfunction/failure over time, yet it’s also widely adopted for clinical outcome prognostication of critical ill patients and those with ARDS [10–12]. Other scoring systems, for instance, Simplified Acute Physiology Score II (SAPS II) [13], Acute Physiology and Chronic Health Evaluation IV (APACHE IV) score [14] and Oxford Acute Severity of Illness Score (OASIS) [15], not intentionally focused on ARDS patients though, have been associated with patient outcomes. However, these scoring systems failed to provide consistent and accurate predictive estimates of the risk of death in patient populations with a specific disease process. In addition, some of the models above require laborious data collection and not easily to be calculated at the bedside. A few previous studies attempted to establish a prognostic model by integrating some predictors of death in ARDS [6–8, 16], but whose predictive power remain controversial. Most of those studies developed their models based on the patients participating in clinical trials and receiving specific treatments, resulting in failure of external validation in real-world patients [17].

Currently, there’s no reliable predictive model available for ARDS patients’ hospital outcomes based on data straight from the bedside and the patients’ actual condition. The primary aim of this study is to develop a clinician-friendly prognostic model incorporating variables that may be relevant to ARDS prognosis and that clinicians could routinely collect and easily calculate to predict risk of in-hospital death in ARDS patients with invasive mechanical ventilation (IMV).

## Methods

### Data sources

All data used in the study was extracted from Medical Information Mart for Intensive Care III (MIMIC-III) database (v1.4) [18], Medical Information Mart for Intensive Care IV (MIMIC-IV) database (v1.0) and eICU Collaborative Research Database (eICU-CRD) [19]. The MIMIC-III includes unidentified health-related data of more than 60,000 ICU stays at Beth Israel Deaconess Medical Center (BIDMC) from June 2001 to October 2012. The MIMIC-IV consists of data of BIDMC from 2008 to 2019. The eICU-CRD is a multicenter database comprising identified health data associated with over 200,000 ICU encounters from 335 units at 208 hospitals located throughout the US between 2014 and 2015. Authors who conduct data acquisition from the databases have completed the course *Protecting Human Research Participants* on the website of National Institutes of Health and obtained the certification (Record ID: 28006489) prior to accession. The three databases have received ethical approval from the Institutional Review Boards (IRBs) at BIDMC and Massachusetts Institute of Technology (MIT). As the databases do not contain identified health information, a waiver of informed consent was included in the approval.

### Study population

All patients in the MIMIC-III, MIMIC-IV and eICU-CRD databases that meet the following criteria will be included in the study. The inclusion criteria were: (I) patients who were 16 years old or more; (II) patients diagnosed as ARDS in the first 48 h of ventilation; (III) receiving invasive ventilation for at least 48 consecutive hours. As onset of ARDS is acute and our cohort is only recently mechanically ventilated patients, patients receiving ventilation through a tracheostomy cannula were excluded. And patients who were extubated or died during the first 48 h were also excluded. Worth noticing, only data of the first ICU admission of the first hospitalization were analyzed. The subjects pooled from MIMIC-III and eICU databases were randomly divided into the training set (70%) to develop the model and the internal validation set (30%) to test the performance of the model. Cohort extracted from MIMIC-IV database according to the same inclusion criteria of MIMIC-III and eICU was served as the external validation cohort. In MIMIC-IV, only data between 2014 and 2019 were included to avoid data duplication with MIMIC-III.

### Data extraction

Structured Query Language (SQL) based on PostgreSQL tools (version 9.6) were used for data extraction. Considering patients from MIMIC-III were admitted before publication of Berlin definition, presence of the ARDS in the first 48 h of ventilation was identified according to the Berlin definition[1] with the SQL code published by PROVE Network Investigators [20]. As the patients from eICU-CRD and MIMIC-IV were admitted at least one year after publication of Berlin definition, we hypothesized patients would be diagnosed as ARDS according to Berlin definition and identified ARDS with International Classification of Diseases (ICD) in the databases. Following demographic data were extracted: age, gender, ethnicity, weight, height, and body mass index (BMI) at the first ICU admission. Medical history included number of comorbidities, asthma, congestive heart failure (CHF), atrial fibrillation (AFIB), chronic renal disease, liver disease, chronic obstructive pulmonary disease, coronary artery disease (CAD), diabetes, hypertension, stroke, and malignancy. Information of diagnosis was also extracted for exploring the etiology of ARDS by classifying ARDS into direct (pulmonary) or indirect (extrapulmonary) ARDS according to previous studies [21, 22]. The usage of vasopressor within the first 24 h of ICU admission was collected. The score including SAPS II in MIMIC-III, APACHE IV in eICU, OASIS and SOFA in the three databases were calculated using the original data. Age, PaO_2_/FiO_2_, and Plateau Pressure Score (APPS) [8] were also calculated. Then, we collected vital signs of the patients within the first 24 h of ICU stay and within the first 24 h of IMV, including heart rate (HR), systolic blood pressure (SBP), diastolic blood pressure (DBP), mean arterial pressure (MAP), temperature, respiratory rate and oxyhemoglobin saturation (SpO_2_). Afterwards, laboratory values within the first 24 h of ICU admission and within the first 24 h of IMV, such as blood routine examination, liver and kidney function, blood glucose, and arterial blood gas (ABG) were extracted. Furthermore, the ventilator parameters within the first 24 h of IMV were also extracted. Owing to the high sampling frequency, we use the maximum, minimum and the mean value when incorporating the characteristics of vital signs, while the related laboratory indicators and ventilator parameters were presented with the maximum and minimum. The data of in-hospital death record were also extracted.

### Statistical analysis

Normally and non-normally distributed continuous variables were presented as the mean ± SD and the median with interquartile range (IQR) respectively. Continuous variables of normal distribution were tested by Kolmogorov-Smirnov test. Student’s t-test, One-way ANOVA, Mann-Whitney U-test or Kruskal-Wallis H-test were used to compare continuous data, if appropriate. Categorical variables were expressed as numbers with percentages and assessed using the Chi-square (χ^2^) test or Fisher’s exact test according to different sample sizes as proper. The Multivariate Imputation by Chained Equations (MICE) package was used for imputations of missing data. Variables whose missing data more than 30% were excluded from the variable selection process.

All patients in the training set were included for variables selection and risk model development. A total of 176 variables were finally entered into the selection process. Least Absolute Shrinkage and Selection Operatory (LASSO) regression was employed to identify the potential strong predictors. Subsequently, variables identified by LASSO regression analysis were entered into the Logistic regression model and those that were consistently statistically significant were further applied to construct the risk model. A nomogram was used to interpret and visualize the risk model.

The risk model was validated in the validation sets. To assess the discrimination of the model, the areas under the receiver operating characteristic curves (AUROCs) for our model and other severity scores were calculated. The calibration slope and the Brier score was constructed for the evaluation of calibration. Decision curve analysis (DCA) [23] was used to determine the clinical usefulness of our model by quantifying the net benefits at different threshold probabilities. The net benefits were calculated by subtracting the proportion of all false-positive patients from the proportion of true-positive patients and by weighing the relative harm of for-going interventions compared with the negative consequences of unnecessary intervention. To assess whether the performance of our model would be affected by the etiology of ARDS and source of patients admitted to hospitals, we further compared the model performance between direct ARDS and indirect ARDS, as well as the model performance between transferred and non-transferred patients.

The data were analyzed with R software (version 4.0.3, R Foundation). A two-tailed *P* < 0.05 was considered statistically significant.

## Results

### Participants and the characteristics of the final cohorts

A total of 1596 patients (535 from MIMIC-III, 521 from MIMIC-IV and 540 from eICU, respectively) were included in the final cohort to be analyzed (Additional File 1: Fig. S1). The subjects pooled from MIMIC-III and eICU were randomly divided into a training cohort (70%, n=752) and an internal validation cohort (30%, n=323). Data from MIMIC-IV was used for external validation. In the training cohort, the overall in hospital mortality was 32.7% and 358 (47.6%) patients developed severe ARDS within the first 48 h of ventilation. Age, comorbidity of liver diseases, comorbidity of malignancy, vasopressor usage at admission, and severity of ARDS are shown significantly different between the deceased patients and the survivors in the training cohort (Table 1). Comparisons upon vital signs, laboratory test results and urine output within both the first 24 h of ICU and the first 24 h of invasive mechanical ventilation between survivors and non-survivors in training cohort are shown in Additional File 1: Tables S1, S2. Differences in ventilator parameters within the first 24 h of ventilation in training cohort are included in Table S2. Characteristics of interval validation and external validation cohort are presented in Table S3 and Table S4, respectively.

**Table 1.** Baseline characteristics of the train cohort comparing survived vs non-survived patients

	Survivor (n=506)	Non-survivor (n=246)	*P*
Age, yr (median [IQR])	58.19 [46.64, 68.06]	64.25 [55.00, 76.92]	<0.001
BMI (median [IQR])	28.53 [24.10, 35.34]	27.64 [23.73, 33.53]	0.187
Gender(male)	284 (56.1%)	138 (56.1%)	>0.999
*Comorbidity, n (%)*			
Asthma	28 (5.5)	10 (4.1)	0.493
CHF	126 (24.9)	71 (28.9)	0.284
AFIB	93 (18.4)	55 (22.4)	0.234
Renal diseases	37 (7.3)	29 (11.8)	0.058
Liver diseases	17 (3.4)	20 (8.1)	0.008
COPD	65 (12.8)	30 (12.2)	0.893
CAD	41 (8.1)	19 (7.7)	0.971
Diabetes	70 (13.8)	32 (13.0)	0.844
Hypertension	120 (23.7)	49 (19.9)	0.281
Stroke	27 (5.3)	16 (6.5)	0.631
Malignancy	53 (10.5)	39 (15.9)	0.046
Number of comorbidities, n (%)			0.297
0	179 (35.4)	66 (26.8)	
1	139 (27.5)	76 (30.9)	
2	93 (18.4)	49 (19.9)	
3	50 (9.9)	35 (14.2)	
4	28 (5.5)	13 (5.3)	
5	13 (2.6)	4 (1.6)	
6	3 (0.6)	2 (0.8)	
7	1 (0.2)	1 (0.4)	
Vasopressor usage	182 (36.0)	111 (45.1)	0.020
ARDS severity, n (%) *			0.003
Mild	60 (11.9)	28 (11.4)	
Moderate	226 (44.7)	80 (32.5)	
Severe	220 (43.5)	138 (56.1)	
Subgroup of ARDS, n (%)			0.643
Direct (pulmonary) ARDS	325 (64.2)	153 (62.2)	
Indirect (extrapulmonary) ARDS	181 (35.6)	93 (37.8)	
*Severity score*			
SAPS II (median [IQR])	41.00 [32.00, 50.00]	49.00 [39.00, 60.00]	<0.001
SOFA (median [IQR])	7.00 [4.00, 9.00]	8.00 [5.00, 11.00]	<0.001
OASIS (median [IQR])	36.00 [31.00, 42.00]	40.00 [34.00, 45.00]	<0.001
APACHE IV (median [IQR])	72.00 [53.00, 91.00]	91.50 [70.75, 114.75]	<0.001
APPS (median [IQR])	6.00 [5.00, 7.00]	6.00 [5.00, 7.00]	<0.001

*BMI* body mass index, *CHF* chronic heart failure, *AFIB* atrial fibrillation, *COPD* chronic obstructive pulmonary disease, *CAD* coronary artery disease,*ARDS* Acute Respiratory Distress Syndromes, *SAPS II* Simplified Acute Physiology Score II, *SOFA* Sequential Organ Failure Assessment, *OASIS* Oxford Acute Severity of Illness Score, *APACHE IV* Acute Physiology and Chronic Health Evaluation IV, *APPS* Age, PaO_2_/FiO_2_, and Plateau Pressure Score

^*^ARDS severity: Mild (200 mmHg < PaO_2_/FiO_2_ ≤300 mmHg); Moderate (100 < PaO_2_/FiO_2_ ≤200 mmHg); Severe (PaO_2_/FiO_2_ ≤100 mmHg)

### Predictors selection and model development

A total of 176 variables measured within the first 24 h of ICU admission and within the first 24 h of IMV were included in the LASSO regression (Additional File 1: Fig. S2). Twelve variables were identified through LASSO regression selection as significant predictors of in-hospital death, including six acquired within the first 24 h after admission to ICU: age, mean of respiratory rate, maximum of international normalized ratio (INR), minimum of red blood cell count, minimum of red blood cell distribution width (RDW) and maximum of alveolo-arterial oxygen difference (AaDO_2_), and six acquired within the first 24 h of IMV: mean of temperature, maximum of lactate, platelet, mean red cell volume (MCV), and minimum of blood urea nitrogen (BUN) and white blood cell count.

**Fig. 1:**
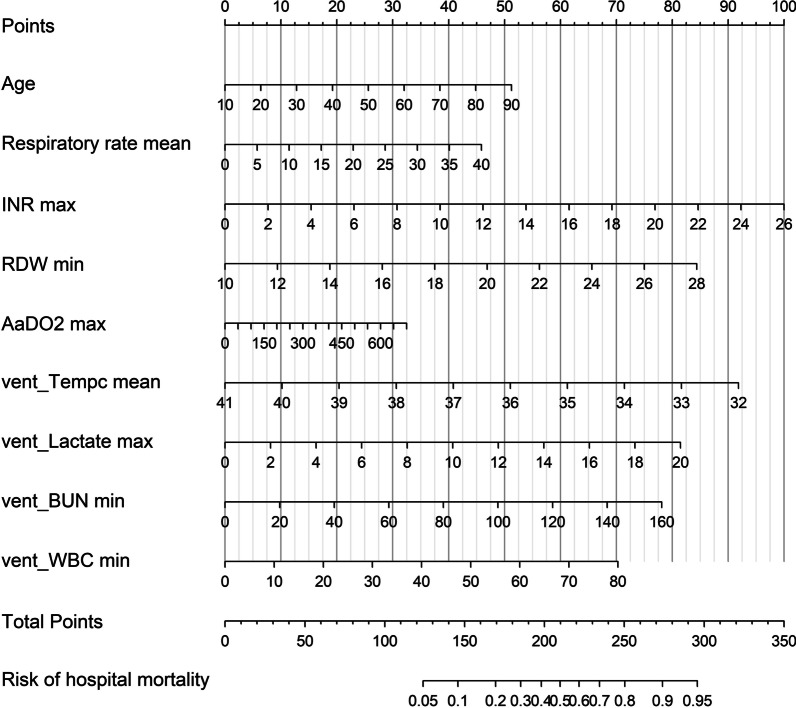
Nomogram to estimate the risk of mortality in ARDS patients. INR international normalized ratio, RDW red blood cell distribution width, AaDO2 alveolo-arterial oxygen difference, Tempc Body temperature, BUN blood urea nitrogen, WBC white blood cell, vent ventilation, max maximum, min minimum. *Note*: Variable name with the prefix of vent means the data was collected within the first 24 h of invasive ventilation

**Fig. 2:**
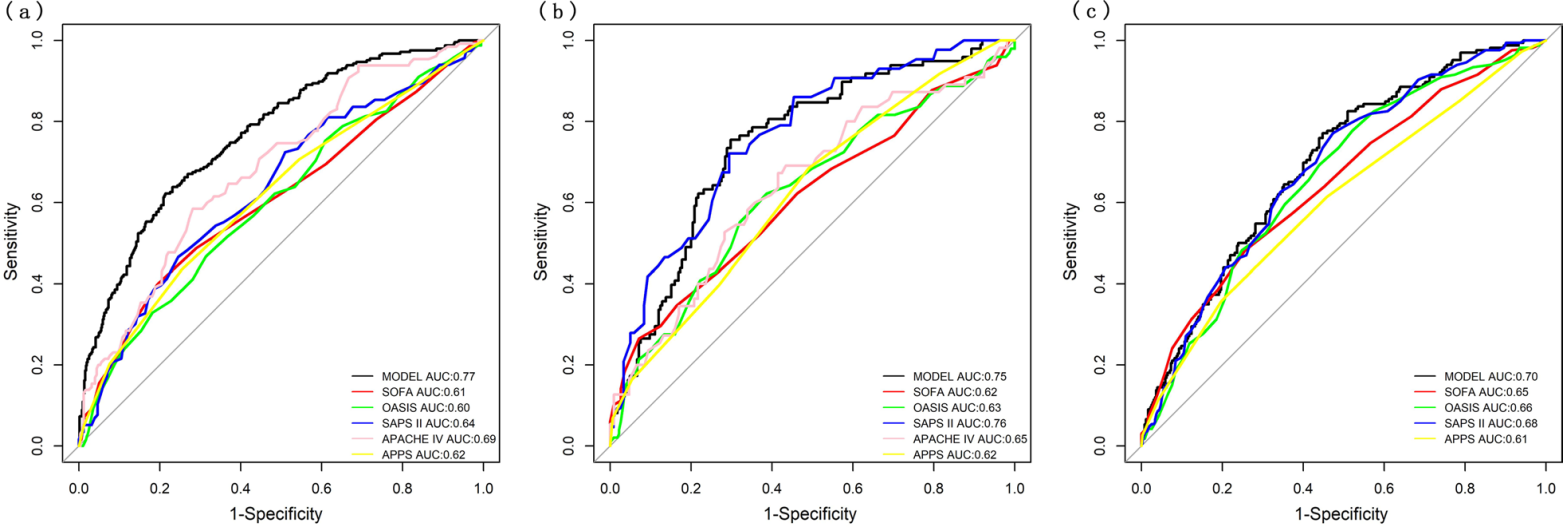
The ROC curves of our model and other severity scores. **a** Training cohort; **b** Internal validation cohort; **c** External validation cohort. *SAPS II* simplified acute physiology score II, *SOFA* sequential organ failure assessment, *OASIS* oxford acute severity of illness score, *APACHE IV* acute physiology and chronic health evaluation IV, *APPS* Age, PaO_2_/FiO_2_, and Plateau Pressure Score

**Fig. 3:**
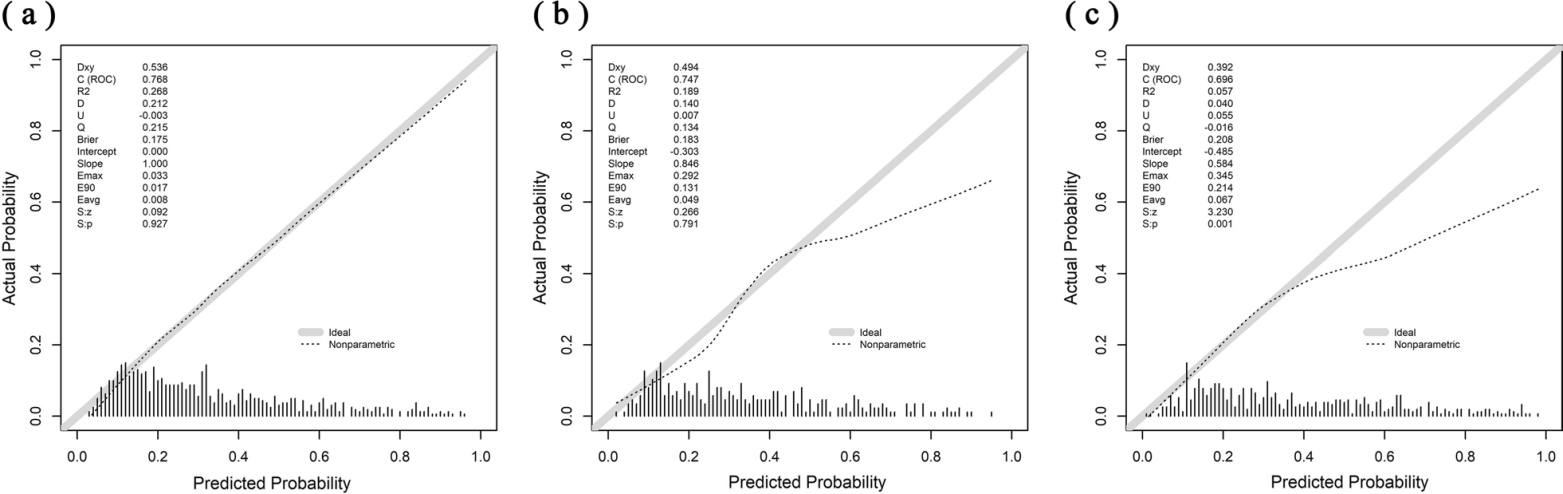
Calibration of our model. **a** Training cohort; **b** Internal validation cohort; **c** External validation cohort

Subsequently, these twelve variables were included in a Logistic regression model and eventually, nine of them outstood as independently statistically significant predictors of in-hospital mortality were included in the risk model. Five variables were ascertained within the first 24 h after ICU admission, including age (OR, 1.02; 95% CI, 1.01–1.03), mean of respiratory rate (OR, 1.04; 95% CI, 1.01–1.08), the maximum of INR (OR, 1.14; 95% CI, 1.03–1.31) and AaDO2 (OR, 1.002; 95% CI, 1.001–1.003), and the minimum of RDW (OR, 1.17; 95% CI, 1.09–1.27). And four factors were measured within the first 24 h after start of IMV, including the mean of temperature (OR, 0.70; 95% CI, 0.57-0.86), the maximum of lactate (OR, 1.15; 95% CI, 1.09–1.22), the minimum of blood urea nitrogen (BUN) (OR, 1.02; 95% CI, 1.01–1.03) and white blood counts (OR, 1.03; 95% CI, 1.01–1.06) (Table 2). Figure 1 presents the nomogram of our model. Our model had a good discrimination (AUC: 0.77; 95% CI: 0.73–0.80) in the training cohort, featuring significant superiority over SOFA, OASIS, SAPS II, APACHE IV and APPS (De Long method, model vs. SOFA: *P* < 0.001; model vs. OASIS: *P* < 0.001; model vs. SAPS II *P* < 0.001; model vs. APACHE IV: *P* < 0.001; model vs. APPS *P* < 0.001) (Fig. 2a) and good calibration (Calibration slope: 1.000, *P* =0.741; Brier score = 0.175) (Fig. 3a).

**Table 2:** Multivariable logistic regression model for predicting hospital mortality in training cohort.

Variables	Odd ratio (95% CI)	*P* value
*Variables on the first 24 hour of ICU admission*		
Age	1.02 (1.01–1.03)	<0.001
Respiratory rate _mean_	1.04 (1.01–1.08)	0.027
INR _max_	1.14 (1.03–1.31)	0.029
RDW _min_	1.17 (1.09–1.27)	<0.001
AaDO_2 max_	1.002 (1.001–1.003)	0.002
*Variables on the first 24 hour of invasive ventilation*		
vent_Tempc _mean_	0.70 (0.57–0.86)	<0.001
vent_Lactate _max_	1.15 (1.09–1.22)	<0.001
vent_BUN _min_	1.02 (1.01–1.03)	<0.001
vent_WBC _min_	1.03 (1.01–1.06)	0.021
constant	205.66	

*INR* international normalized ratio, *RDW* red blood cell distribution width, *AaDO*_*2*_ alveolo-arterial oxygen difference, *Tempc* Body temperature, *BUN* blood urea nitrogen, *WBC* white blood cell, *vent* ventilation, *max* maximum, *min* minimum. Variable name with the prefix of *vent* means the data was collected within 24 h of invasive ventilation

### Model performance

**Fig. 4:**
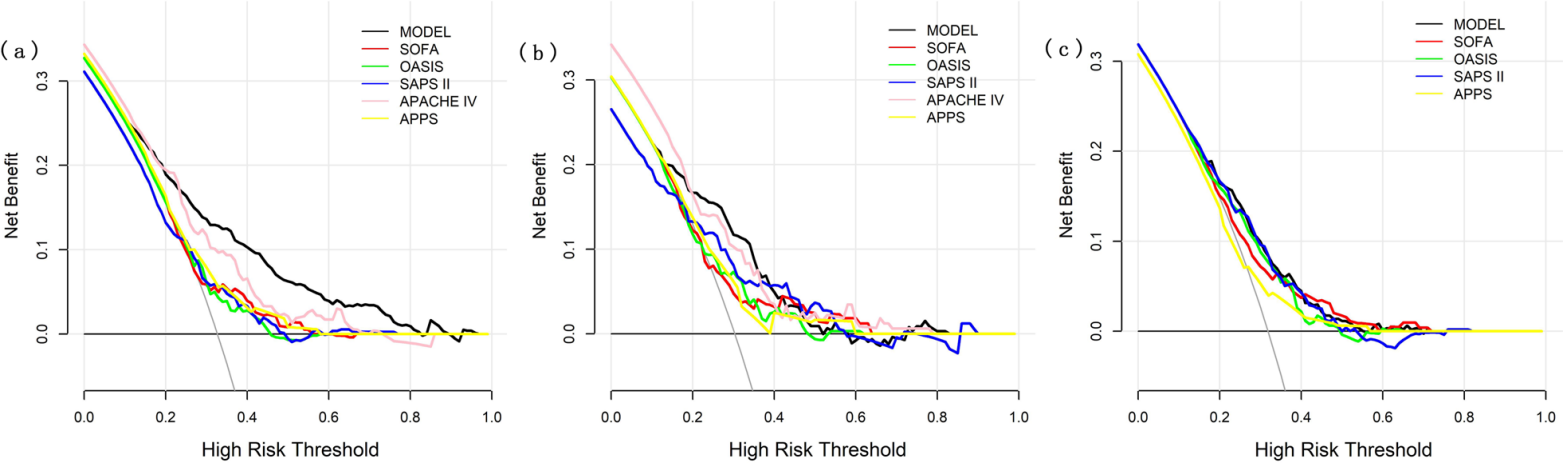
Decision curve analysis of our model and other severity scores. **a** Training cohort; **b** Internal validation cohort; **c** External validation cohort *SAPS II* simplified acute physiology score II, *SOFA* sequential organ failure assessment, *OASIS* oxford acute severity of illness score, *APACHE IV* acute physiology and chronic health evaluation IV, *APPS* Age, PaO_2_/FiO_2_, and Plateau Pressure Score

Discrimination and calibration of the model were evaluated in both internal and external validation cohorts. Our model remained well-discriminated in the internal validation cohort (AUC: 0.75, 95% CI: 0.69–0.80), which was greater than APACHE IV, SOFA, OASIS and APPS (AUC: APACHE IV 0.65; SOFA 0.62; OASIS 0.63; APPS 0.62; Fig. 2b). Although the discrimination was lower than that of SAPS II (AUC: 0.76), no statistical significance was observed (De Long method, model vs. SAPS II *P* =0.49). In addition, a considerable calibration was showed in our model (Calibration slope: 0.846; Brier score = 0.183) (Fig. 3b). In terms of predicting in-hospital mortality, the DCA results of our model, SAPS II, OASIS, SOFA, APACHE IV and APPS were shown in Fig. 4. DCA of our model indicates that if the threshold probability of a patient is set between 20% and 60%, then the use of our model is more beneficial to patients compared with the extreme situation of mortality of ARDS in all patients or none. These findings suggest that our model provides a higher net benefit across a reasonably wide range of threshold probabilities for predicting mortality of ARDS, and thus has good clinical utility. The net benefit of our model was also better than the SAPS II, OASIS, SOFA, APACHE IV and APPS in this range. We further externally validated our model in a cohort of MIMIC-IV and our model outperformed the SAPS II, OASIS, SOFA and APPS (Fig. 2c). The AUC of our model in external validation was 0.70 (95% CI: 0.65–0.74) with a brier score of 0.208 (Fig. 3c). The performance of our model in patients of direct ARDS and indirect ARDS is shown in Additional File 1: Fig. S3. The performance of our model in patients of transferred and non-transferred from other hospitals is shown in Additional File 1: Fig. S4.

## Discussion

In this study, we developed and externally validated a clinical risk model and constructed a nomogram to predict the mortality of ventilated ARDS patients with LASSO method, which is suitable for the regression of high-dimensional data. Our model shows a moderate performance in predicting in-hospital mortality specifically for ventilated ARDS patients. Only nine simple variables routinely recorded in clinical practice are required for the prediction of in-hospital mortality in our model. Hence, our model can be easily implemented with the nomogram. In the validation cohort, the discrimination of our model was comparable to SAPS II and was significantly better APACHE IV, SOFA and OASIS.

Mortality prediction in ICU patients has been widely investigated in recent years, but the general ICU severity scores were not sufficient for predicting mortality in the population of invasively ventilated ARDS patients accurately and reliably. Several studies evaluated scoring systems (including APACHE IV, SOFA, APACHE II etc.) in ARDS patients, reporting poor to moderate discrimination for these scores [24–26]. In our study, the AUC of APACHE IV, SOFA and OASIS on predicting hospital mortality of ventilated ARDS were < 0.65 in internal validation cohort or external validation cohort, suggesting a low discriminatory power. Efforts on predicting mortality in patients with ARDS had been made by investigators. The APPS score, with a 9-point scale, incorporated the variables of age, plateau pressure and arterial oxygen partial pressure to fractional inspired oxygen ratio (PaO_2_/FiO_2_) reached an AUC of 0.80 [8], but its AUC significantly decreased to 0.62 in an independent cohort [17], which is similar to the performance in our cohort. Zhao and colleagues constructed a model combining age, APACHE III, surfactant protein D (SP-D) and interleukin-8 (IL-8) for the prediction of ARDS mortality based on ALVEOLI cohort [7] and the performance in two external cohorts (FACTT and VALID) [27] were comparable to our model. However, neither SP-D nor IL-8 is prosaically tested in clinics, as well as the complicated calculation of APACHE III score consisting of a multitude of variables, turning the timely clinical decision making into a major challenge for intensivists confronting ARDS patients. Huang et al [28] constructed a model based on Random Forest algorithm showed better performance in external validation compared to our model (Random Forest vs. Logistic: 0.74 vs. 0.70) but included more variables (twelve) than ours. Generally, the performance of a scoring system improves as factors increase. In addition, Huang et al did not provide a visualized tool for evaluating the risk of mortality (nomogram or scoring system), which limits its clinical practicability. A systematic review [29] showed that regarding clinical prediction model with binary outcome, so far, no evidence supports that machine learning algorithm performs better than traditional Logistic regression in terms of prediction ability.

Therefore, we aimed at developing a model with a handful of routinely checked variables for mortality prediction for the ventilated ARDS patients that can be easily worked out by the bedside. Nine independent variables from 176 clinical features were finally identified using LASSO method and subsequent Logistic regression by examining the predictor-outcome association.

Interestingly, increased body temperature within the first 24 h of ventilation was negatively related to death in our model, which is consistent with results noted in two published studies [30, 31]. Although a prospective clinical trial reported that aggressive fever suppression group showed a higher mortality compared to permissive group based on a cohort of critically ill trauma patients [32], the underlying mechanism remained unknown.

In addition, RDW within the first 24 h of admission was included in our model as an important risk factor. RDW is a measurement of the amount of red blood cell variation in volume and size, which has been recently found to be abnormally increased in COVID-19 [33] and an independent risk factor for the development and outcome of ARDS [34–36]. High lactate level is considered as a nonspecific marker for tissue hypoxemia, which has been reported as a predictive factor for a poor outcome among critical ill patients [37–39]. Another crucial predictive factor in our model is INR, which, however, was not included in existing risk scores. A previous study reported that INR was associated with hospital mortality of ARDS [40]. INR was also found to be significantly higher in ARDS patients with diffuse alveolar damage (DAD) compared with those without DAD [41]. Other variables such as advanced age, high respiratory rate, increased AaDO_2_, high BUN, and hyperleukocytosis were found to be associated with ARDS events or outcome of ARDS [8, 11, 42–44].

Our model is simple for calculation and easy to use with the nomogram, and has robust discrimination and calibration. Besides, we carried out the decision curve analysis to explore the clinical use of our model, and there was a considerable range of alternative threshold probability. Also, our model was constructed based on multicenter data and the external validation was also performed, which improved its generalizability. Moreover, the predictors that we adopted are no extraordinary data regularly obtained from the patients, enabling ICU caregivers to predict the mortality risk of ventilated ARDS patients and improve clinical decision-making right at the bedside. A previous study which secondly analyzed the VALID trial, reported that direct and indirect ARDS have distinct features that may differentially affect risk prediction and clinical outcomes, while the discrimination of our model seems to be stable and did not affect by direct or indirect etiology [22]. The discriminations of our model seem to be not affected by direct or indirect etiology (direct: AUC: 0.69, 95% CI: 0.63–0.75) (indirect: AUC: 0.70, 95% CI: 0.62–0.78) (*P*=0.858). Whether the predictors of hospital mortality differ among different etiologies of ARDS is still unknown and studies focus on this are valuable.

Several limitations need to be acknowledged. First of all, as the study was retrospective and observationally designed, several inherent limitations like selection bias, loss to follow up and the presence of confounding factors cannot be avoided. Further prospective studies are needed to evaluate the effectiveness of our model. Secondly, some of the variables were excluded for the missing data although previous research has shown that they might be associated with mortality of ARDS patients, such as albumin [45], hepatic function [46, 47] and neutrophil-to-lymphocyte ratio (NLR) [48]. Thirdly, some studies reported that echocardiographic findings were associated with the outcomes of ARDS and COVID-19 [49–51], but we did not include relevant variables because information of echocardiographic findings was only available in a small part of patients in MIMIC-III database but not recorded in eICU and MIMIC-IV databases. Future study including echocardiographic findings is appreciated. Last but not least, similar to previous risk sores, the results of external validation indicated that the discrimination and calibration were decreased compared with that of the training cohort and internal validation cohort, with an overestimate of hospital mortality, which would be owing to the changing strategy of managing ARDS over a long period. Further optimization with more updated data of ARDS patients (recent five years) would be appreciated.

## Conclusions

A risk score based on routinely collected variables at the start of admission to ICU and invasive ventilation can predict mortality of ventilated ARDS patients, with a moderate performance. And further evaluation of our model is required.

## Supplementary Information

**Additional file 1. Table S1.** Vital signs and Laboratory findings within the first 24 hours of ICU admission among survivors and non-survivors in training cohort. **Table S2.** Vital signs, laboratory findings and ventilator settings within the first 24 hours of ventilation among survivors and non-survivors in training cohort. **Table S3.** Characteristics of the internal validation cohort comparing survived vs non-survived patients. **Table S4**. Characteristics of the external validation cohort comparing survived vs non-survived patients. **Figure S1.** The detailed process of data extraction. **Figure S2.** Feature selection using the least absolute shrinkage and selection operator (LASSO) binary logistic regression model. **Figure S3.** The ROC curves of our model validated in cohort of direct ARDS and indirect ARDS. **Figure S4.** The ROC curves of our model validated in cohort of transferred and non-transferred.

## Data Availability

The datasets are available in the website of PhysioNet. MIMIC-III: https://physionet.org/content/mimiciii/1.4/; MIMIC-IV: https://physionet.org/content/mimiciv/1.0/; eICU: https://physionet.org/content/eicu-crd/2.0/ The above mentioned links are the direct persistent links to the datasets and researchers need to completed the course Protecting Human Research Participants on the website of National Institutes of Health and obtained the certification prior to accession.

## Abbreviations

ARDS: Acute respiratory distress syndrome;
ICU: Intensive care unit
IMV: Invasive mechanical ventilation
MIMIC: Medical information mart for intensive care
eICU-CRD: eICU Collaborative research database
INR: International normalized ratio
RDW: Blood cell distribution width
AaDO_2_: Alveolo-arterial oxygen difference
BUN: Blood urea nitrogen
WBC: White blood cell
SAPS II: Simplified acute physiology score II
APACHE II/III/IV: Acute physiology and chronic health evaluation II/III/IV
OASIS: Oxford acute severity of illness score
SOFA: Sequential organ failure assessment
APPS: Age
PaO_2_/FiO_2_: Plateau pressure score
