# Supplementary material for: Development and validation of a clinical risk model to predict the hospital mortality in ventilated patients with acute respiratory distress syndrome: a population-based study
Source: BMC Pulm Med. 2022 Jul 11;22:268. doi: 10.1186/s12890-022-02057-0 (PMC9277886; doi:10.1186/s12890-022-02057-0)
Supplement: Supplementary file 1 — Additional file 1. Table S1. Vital signs and Laboratory findings within the first 24 hours of ICU admission among survivors and non-survivors in training cohort. Table S2. Vital signs, laboratory findings and ventilator settings within the first 24 hours of ventilation among survivors and non-survivors in training cohort. Table S3. Characteristics of the internal validation cohort comparing survived vs non-survived patients. Table S4. Characteristics of the external validation cohort comparing survived vs non-survived patients. Figure S1. The detailed process of data extraction. Figure S2. Feature selection using the least absolute shrinkage and selection operator (LASSO) binary logistic regression model. Figure S3. The ROC curves of our model validated in cohort of direct ARDS and indirect ARDS. Figure S4. The ROC curves of our model validated in cohort of transferred and non-transferred. [file 12890_2022_2057_MOESM1_ESM.docx]

**Additional file 1**

**Table S1. Vital signs and Laboratory findings within the first 24 hours of ICU admission among survivors and non-survivors in training cohort**

|  | Survivor  (n=506) | Non-survivor  (n=246) | *P* |
| --- | --- | --- | --- |
| heartrate_min (times/min, median [IQR]) | 78.00 [66.00, 90.00] | 75.00 [65.00, 90.00] | 0.366 |
| heartrate_max (times/min, median [IQR]) | 117.00 [101.75, 131.25] | 116.00 [103.00, 134.00] | 0.816 |
| heartrate_mean (times/min, median [IQR]) | 94.22 [82.03, 107.20] | 94.28 [81.22, 106.82] | 0.871 |
| sysbp_min (mmHg, median [IQR]) | 84.00 [74.00, 94.00] | 79.00 [72.00, 93.00] | 0.032 |
| sysbp_max (mmHg, median [IQR]) | 149.00 [135.00, 168.00] | 151.00 [134.00, 169.00] | 0.771 |
| sysbp_mean (mmHg, median [IQR]) | 111.78 [103.45, 123.65] | 111.22 [102.39, 122.03] | 0.289 |
| diasbp_min (mmHg, median [IQR]) | 44.00 [37.00, 51.00] | 41.00 [33.00, 48.00] | 0.001 |
| diasbp_max (mmHg, median [IQR]) | 86.00 [77.00, 97.00] | 86.00 [74.00, 102.00] | 0.568 |
| diasbp_mean (mmHg, median [IQR]) | 61.37 [55.97, 67.79] | 59.41 [53.00, 66.16] | 0.013 |
| meanbp_min (mmHg, median [IQR]) | 57.00 [49.00, 64.00] | 54.00 [47.00, 62.83] | 0.015 |
| meanbp_max (mmHg, median [IQR]) | 105.00 [94.00, 122.00] | 106.00 [92.00, 121.00] | 0.658 |
| meanbp_mean (mmHg, median [IQR]) | 76.38 [71.35, 84.27] | 74.88 [68.74, 83.60] | 0.023 |
| resprate_min (times/min, median [IQR]) | 14.00 [11.00, 17.00] | 14.00 [11.00, 18.00] | 0.193 |
| resprate_max (times/min, median [IQR]) | 31.00 [26.00, 37.00] | 33.00 [28.00, 38.00] | 0.055 |
| resprate_mean (times/min, median [IQR]) | 21.43 [18.02, 25.50] | 23.05 [19.19, 26.58] | 0.004 |
| tempc_min (℃,median [IQR]) | 36.50 [35.94, 36.83] | 36.11 [35.50, 36.60] | <0.001 |
| tempc_max (℃,median [IQR]) | 37.90 [37.28, 38.60] | 37.61 [37.00, 38.35] | <0.001 |
| tempc_mean (℃,median [IQR]) | 37.14 [36.76, 37.63] | 36.86 [36.46, 37.38] | <0.001 |
| spo2_min (%,median [IQR]) | 90.00 [85.00, 93.00] | 89.00 [84.00, 92.00] | 0.210 |
| spo2_max (%,median [IQR]) | 100.00 [100.00, 100.00] | 100.00 [100.00, 100.00] | 0.902 |
| spo2_mean (%,median [IQR]) | 96.55 [94.97, 98.00] | 96.23 [94.80, 97.90] | 0.346 |
| bicarbonate_min (mmol/L, median [IQR]) | 21.00 [18.00, 25.00] | 20.00 [16.00, 23.00] | <0.001 |
| bicarbonate_max (mmol/L, median [IQR]) | 24.00 [22.00, 28.00] | 23.00 [21.00, 27.00] | 0.008 |
| bilirubin_min (mg/dL, median [IQR]) | 0.65 [0.40, 1.00] | 0.70 [0.40, 2.00] | 0.005 |
| bilirubin_max (mg/dL, median [IQR]) | 0.70 [0.50, 1.30] | 0.90 [0.50, 2.75] | 0.001 |
| creatinine_min (mg/dL, median [IQR]) | 0.91 [0.67, 1.40] | 1.20 [0.80, 1.94] | <0.001 |
| creatinine_max (mg/dL, median [IQR]) | 1.20 [0.80, 2.00] | 1.50 [0.98, 2.36] | <0.001 |
| chloride_min (mmol/L, median [IQR]) | 103.00 [99.00, 107.00] | 102.00 [98.00, 107.00] | 0.395 |
| chloride_max (mmol/L, median [IQR]) | 107.00 [103.00, 112.00] | 107.00 [102.00, 111.00] | 0.671 |
| glucose_min (mg/dL, median [IQR]) | 106.00 [89.00, 132.00] | 107.00 [83.25, 135.00] | 0.837 |
| glucose_max (mg/dL, median [IQR]) | 177.00 [137.00, 236.75] | 187.50 [147.25, 248.00] | 0.028 |
| hematocrit_min (%,median [IQR]) | 30.85 [26.30, 36.08] | 29.80 [25.00, 34.60] | 0.017 |
| hematocrit_max (%,median [IQR]) | 35.90 [31.35, 41.00] | 34.20 [30.40, 39.50] | 0.019 |
| hemoglobin_min (g/dL,mean(SD)) | 10.47±2.23 | 9.85±2.41 | 0.001 |
| hemoglobin_max (g/dL, median [IQR]) | 12.00 [10.40, 13.57] | 11.20 [10.00, 13.00] | 0.006 |
| lactate_min (mmol/L,median [IQR]) | 1.40 [1.00, 2.10] | 1.80 [1.20, 2.80] | <0.001 |
| lactate_max (mmol/L,median [IQR]) | 2.40 [1.40, 4.30] | 3.15 [1.80, 6.70] | <0.001 |
| platelet_min (10^9/L,median [IQR]) | 183.00 [116.75, 247.25] | 148.00 [77.00, 228.00] | 0.001 |
| platelet_max (10^9/L,median [IQR]) | 220.50 [158.00, 294.25] | 192.00 [120.00, 281.00] | 0.002 |
| potassium_min (mmol/L,median [IQR]) | 3.70 [3.30, 4.00] | 3.70 [3.30, 4.20] | 0.406 |
| potassium_max (mmol/L,median [IQR]) | 4.40 [3.90, 4.90] | 4.50 [4.00, 5.30] | 0.018 |
| inr_min (median [IQR]) | 1.20 [1.10, 1.40] | 1.30 [1.11, 1.70] | <0.001 |
| inr_max (median [IQR]) | 1.30 [1.20, 1.70] | 1.60 [1.20, 2.30] | <0.001 |
| pt_min (sec, median [IQR]) | 13.90 [12.80, 15.40] | 14.60 [13.30, 17.65] | <0.001 |
| pt_max (sec, median [IQR]) | 14.90 [13.50, 17.10] | 16.70 [13.90, 22.95] | <0.001 |
| sodium_min (mmol/L, median [IQR]) | 137.00 [134.00, 140.00] | 137.00 [134.00, 140.00] | 0.468 |
| sodium_max (mmol/L, median [IQR]) | 141.00 [138.00, 144.00] | 140.00 [137.00, 144.00] | 0.912 |
| bun_min (mg/dL, median [IQR]) | 18.00 [12.00, 30.00] | 27.00 [17.00, 42.00] | <0.001 |
| bun_max (mg/dL, median [IQR]) | 23.00 [16.00, 36.25] | 33.00 [21.00, 48.00] | <0.001 |
| wbc_min (10^9/L, median [IQR]) | 10.40 [6.65, 14.72] | 11.70 [6.70, 15.40] | 0.142 |
| wbc_max (10^9/L, median [IQR]) | 13.85 [9.70, 19.22] | 15.37 [10.20, 19.80] | 0.149 |
| ca_t_min (mmol/L, median [IQR]) | 7.80 [7.20, 8.40] | 7.70 [7.10, 8.20] | 0.318 |
| ca_t_max (mmol/L, median [IQR]) | 8.40 [7.80, 8.90] | 8.30 [7.80, 8.90] | 0.815 |
| rbc_min (10^12/L, median [IQR]) | 3.45 [2.97, 4.03] | 3.32 [2.77, 3.87] | 0.002 |
| rbc_max (10^12/L, median [IQR]) | 3.97 [3.46, 4.54] | 3.76 [3.25, 4.33] | 0.001 |
| mch_min (pg, median [IQR]) | 29.90 [28.45, 31.10] | 30.20 [28.50, 31.60] | 0.149 |
| mch_max (pg, median [IQR]) | 30.50 [29.00, 31.80] | 30.90 [29.00, 32.20] | 0.096 |
| mchc_min (%, median [IQR]) | 33.00 [31.90, 33.90] | 32.70 [31.60, 33.70] | 0.023 |
| mchc_max (%, median [IQR]) | 33.80 [32.70, 34.90] | 33.50 [32.20, 34.70] | 0.101 |
| mcv_min (fL, median [IQR]) | 89.00 [85.00, 93.00] | 90.00 [86.00, 95.00] | 0.02 |
| mcv_max (fL, median [IQR]) | 91.00 [87.00, 95.00] | 93.00 [88.00, 98.00] | 0.002 |
| rdw_min (%, median [IQR]) | 14.30 [13.40, 15.70] | 15.20 [14.00, 17.10] | <0.001 |
| rdw_max (%, median [IQR]) | 14.70 [13.75, 16.00] | 15.50 [14.30, 17.98] | <0.001 |
| po2_min (mmHg, median [IQR]) | 66.00 [55.00, 80.00] | 65.00 [54.00, 76.95] | 0.255 |
| po2_max (mmHg, median [IQR]) | 163.00 [106.00, 242.00] | 166.00 [106.50, 253.50] | 0.805 |
| pco2_min (mmHg, median [IQR]) | 36.00 [31.00, 42.00] | 34.00 [28.00, 40.00] | <0.001 |
| pco2_max (mmHg, median [IQR]) | 49.10 [41.80, 59.00] | 48.00 [40.00, 60.00] | 0.218 |
| aado2_min (mmHg, median [IQR]) | 238.48 [146.31, 420.46] | 309.55 [172.10, 464.00] | 0.004 |
| aado2_max (mmHg, median [IQR]) | 427.00 [243.70, 570.25] | 495.00 [312.49, 593.64] | 0.001 |
| ph_min (median [IQR]) | 7.28 [7.20, 7.35] | 7.25 [7.16, 7.36] | 0.061 |
| ph_max (median [IQR]) | 7.41 [7.37, 7.46] | 7.41 [7.34, 7.47] | 0.594 |
| baseexcess_min (mmol/L, median [IQR]) | -4.00 [-9.00, 0.10] | -5.25 [-11.00, 0.00] | 0.011 |
| baseexcess_max (mmol/L, median [IQR]) | 0.00 [-2.00, 3.50] | 0.00 [-3.00, 2.60] | 0.031 |
| albumin_min, g/dL (median [IQR]) | 2.70 [2.30, 3.10] | 2.50 [1.90, 2.90] | <0.001 |
| albumin_max, g/dL (median [IQR]) | 2.90 [2.40, 3.30] | 2.70 [2.30, 3.20] | 0.02 |
| bilirubin_min, g/dL (median [IQR]) | 0.65 [0.40, 1.00] | 0.70 [0.40, 2.00] | 0.005 |
| bilirubin_max, g/dL (median [IQR]) | 0.70 [0.50, 1.30] | 0.90 [0.50, 2.75] | 0.001 |
| ALT_min, IU/L (median [IQR]) | 30.0 [19.0, 57.0] | 34.0 [19.0, 78.5] | 0.155 |
| ALT_max, IU/L (median [IQR]) | 35.0 [22.0, 71.0] | 44.0 [21.5, 121] | 0.043 |
| AST_min, IU/L (median [IQR]) | 43.0 [24.0, 76.8] | 61.0 [29.5, 124] | 0.001 |
| AST_max, IU/L (median [IQR]) | 51.0 [27.0, 116] | 74.0 [36.0, 229] | <0.001 |
| ALP_min, IU/L (median [IQR]) | 78.0 [57.0, 107] | 88.0 [65.0, 128] | 0.004 |
| ALP_max, IU/L (median [IQR]) | 83.0 [62.0, 115] | 99.0 [74.0, 141] | <0.001 |
| urine output (mL, median [IQR]) | 1716.50 [922.25, 2691.50] | 1198.00 [653.00, 2050.00] | <0.001 |

*sysbp* systolic blood pressure, *diasbp* diastolic blood pressure, *meanbp* mean blood pressure, *resprate* respiratary rate, *tempc* temperature, *bun* blood urea nitrogen, *wbc* white blood cell, *INR* international normalized ratio, *Spo2* oxyhemoglobin saturation, *pt* prothrombin time, *rbc* red blood cell, *mch* mean hemoglobin content of red blood cells, *mchc* mean corpuscular hemoglobin concentration, *mcv* mean corpuscular volume, *rdw* red blood cell distribution width, *aado2* alveolar-arterial oxygen gradient, *po2* oxygen partial pressure, *pco2* partial pressure of carbon dioxide, *ca_t* total calcium, *ALT* Alanine Aminotransferase, *AST* Asparate Aminotransferase, *ALP* Alkaline Phosphatase, *max* maximum, *min* minimum

Note: Variable name with the prefix of vent means the data was collected at the beginning of invasive ventilation.

**Table S2. Vital signs, laboratory findings and ventilator settings within the first 24 hours of ventilation among survivors and non-survivors in training cohort**

|  | Survivor  (n=506) | Non-survivor  (n=246) | *P* |
| --- | --- | --- | --- |
| vent_heartrate_min (times/min, median [IQR]) | 76.00 [64.00, 89.00] | 73.00 [63.25, 87.00] | 0.264 |
| vent_heartrate_max (times/min, median [IQR]) | 116.00 [101.00, 131.00] | 117.50 [101.25, 134.00] | 0.462 |
| vent_heartrate_mean (times/min, median [IQR]) | 92.92 [80.40, 105.85] | 92.35 [80.21, 105.80] | 0.901 |
| vent_sysbp_min (mmHg, median [IQR]) | 82.00 [73.00, 92.00] | 78.00 [69.00, 89.00] | 0.003 |
| vent_sysbp_max (mmHg, median [IQR]) | 148.50 [134.00, 167.00] | 150.00 [133.00, 169.00] | 0.585 |
| vent_sysbp_mean (mmHg, median [IQR]) | 110.15 [102.60, 119.39] | 109.10 [101.66, 119.58] | 0.389 |
| vent_diasbp_min (mmHg, median [IQR]) | 44.00 [37.00, 50.00] | 41.00 [34.00, 47.00] | <0.001 |
| vent_diasbp_max (mmHg, median [IQR]) | 85.00 [75.25, 96.00] | 83.00 [72.00, 98.00] | 0.414 |
| vent_diasbp_mean (mmHg, median [IQR]) | 60.75 [55.76, 66.66] | 58.10 [52.39, 64.34] | 0.001 |
| vent_meanbp_min (mmHg, median [IQR]) | 56.50 [49.00, 63.00] | 54.00 [46.00, 60.00] | 0.001 |
| vent_meanbp_max (mmHg, median [IQR]) | 104.83 [93.00, 120.92] | 104.00 [92.00, 121.00] | 0.593 |
| vent_meanbp_mean (mmHg, median [IQR]) | 75.74 [71.07, 82.55] | 73.89 [68.89, 81.86] | 0.023 |
| vent_resprate_min (times/min, median [IQR]) | 13.00 [10.00, 16.00] | 14.00 [10.00, 17.00] | 0.559 |
| vent_resprate_max (times/min, median [IQR]) | 30.00 [25.00, 35.00] | 31.00 [26.00, 36.00] | 0.041 |
| vent_resprate_mean (times/min, median [IQR]) | 20.70 [17.41, 24.84] | 21.85 [18.56, 25.81] | 0.009 |
| vent_tempc_min (℃, median [IQR]) | 36.50 [35.94, 36.90] | 36.20 [35.54, 36.62] | <0.001 |
| vent_tempc_max (℃, median [IQR]) | 38.00 [37.40, 38.60] | 37.70 [37.17, 38.39] | 0.001 |
| vent_tempc_mean (℃, median [IQR]) | 37.21 [36.82, 37.64] | 36.99 [36.52, 37.42] | <0.001 |
| vent_spo2_min (%, median [IQR]) | 91.00 [86.00, 93.00] | 90.00 [84.75, 93.00] | 0.32 |
| vent_spo2_max (%, median [IQR]) | 100.00 [100.00, 100.00] | 100.00 [100.00, 100.00] | 0.743 |
| vent_spo2_mean (%, median [IQR]) | 96.96 [95.57, 98.20] | 96.62 [95.05, 98.21] | 0.324 |
| vent_po2_min (mmHg, median [IQR]) | 67.00 [56.00, 82.00] | 65.00 [54.00, 79.62] | 0.280 |
| vent_po2_max (mmHg, median [IQR]) | 174.00 [116.00, 247.00] | 162.50 [110.85, 255.25] | 0.666 |
| vent_pco2_min (mmHg, median [IQR]) | 36.00 [31.95, 42.00] | 34.00 [29.00, 39.27] | <0.001 |
| vent_pco2_max (mmHg, median [IQR]) | 50.00 [42.00, 60.00] | 49.35 [41.15, 61.50] | 0.598 |
| vent_aado2_min_firstvent (mmHg, median [IQR]) | 249.94 [160.20, 420.24] | 314.40 [186.11, 464.00] | 0.001 |
| vent_aado2_max_firstvent (mmHg, median [IQR]) | 435.96 [269.90, 574.57] | 497.51 [321.08, 586.99] | 0.002 |
| vent_ph_min (median [IQR]) | 7.27 [7.20, 7.35] | 7.24 [7.15, 7.33] | 0.009 |
| vent_ph_max (median [IQR]) | 7.41 [7.36, 7.46] | 7.41 [7.34, 7.47] | 0.676 |
| vent_baseexcess_min(mmol/L, median [IQR]) | -4.00 [-8.70, 0.40] | -5.00 [-11.00, 0.00] | 0.004 |
| vent_baseexcess_max(mmol/L, median [IQR]) | 0.30 [-2.00, 4.00] | 0.00 [-3.00, 3.00] | 0.018 |
| vent_tidal_volume_min (mL, median [IQR]) | 440.00 [375.00, 500.00] | 443.00 [369.25, 500.00] | 0.924 |
| vent_tidal_volume_max (mL, median [IQR]) | 600.00 [520.00, 700.00] | 600.00 [519.25, 697.50] | 0.572 |
| vent_peep_min (cmH_2_O, median [IQR]) | 5.00 [5.00, 8.00] | 5.00 [5.00, 8.00] | 0.774 |
| vent_peep_max (cmH_2_O, median [IQR]) | 10.00 [5.00, 12.75] | 10.00 [5.00, 14.00] | 0.281 |
| vent_plateau_pressure_min (cmH_2_O, median [IQR]) | 20.00 [16.00, 24.00] | 20.00 [17.00, 25.00] | 0.261 |
| vent_plateau_pressure_max (cmH_2_O, median [IQR]) | 27.00 [22.00, 32.00] | 27.50 [23.00, 33.00] | 0.278 |
| vent_peak_insp_pressure_min (cmH_2_O, median [IQR]) | 22.00 [17.00, 26.00] | 21.00 [16.00, 26.00] | 0.451 |
| vent_peak_insp_pressure_max (cmH_2_O, median [IQR]) | 33.00 [28.00, 38.00] | 34.00 [28.00, 39.00] | 0.342 |
| vent_resp_rate_set_min (times/min, median [IQR]) | 15.00 [12.00, 19.00] | 14.00 [12.00, 20.00] | 0.773 |
| vent_resp_rate_set_max (times/min, median [IQR]) | 24.00 [18.00, 30.00] | 25.00 [20.00, 30.00] | 0.040 |
| vent_fio2_min (%, median [IQR]) | 50.00 [40.00, 50.00] | 50.00 [40.00, 60.00] | 0.004 |
| vent_fio2_max (%, median [IQR]) | 80.00 [60.00, 100.00] | 100.00 [60.00, 100.00] | 0.015 |
| vent_bicarbonate_min(mmol/L, median [IQR]) | 21.00 [18.00, 25.00] | 20.00 [16.00, 24.00] | <0.001 |
| vent_bicarbonate_max(mmol/L, median [IQR]) | 24.00 [21.25, 28.00] | 23.00 [21.00, 27.00] | 0.002 |
| vent_creatinine_min(mg/dL, median [IQR]) | 0.90 [0.64, 1.46] | 1.30 [0.80, 2.00] | <0.001 |
| vent_creatinine_max(mg/dL, median [IQR]) | 1.19 [0.80, 2.00] | 1.60 [1.00, 2.36] | <0.001 |
| vent_chloride_min(mmol/L, median [IQR]) | 103.50 [99.00, 108.00] | 103.00 [99.00, 107.00] | 0.575 |
| vent_chloride_max(mmol/L, median [IQR]) | 108.00 [103.00, 112.00] | 107.00 [104.00, 111.00] | 0.739 |
| vent_glucose_min(mg/dL, median [IQR]) | 106.00 [87.00, 132.00] | 108.00 [85.25, 135.00] | 0.715 |
| vent_glucose_max(mg/dL, median [IQR]) | 177.00 [137.00, 238.25] | 192.00 [152.25, 248.00] | 0.009 |
| vent_hematocrit_min(%,median [IQR]) | 30.25 [25.80, 35.40] | 29.50 [24.60, 34.00] | 0.035 |
| vent_hematocrit_max(%,median [IQR]) | 35.00 [30.98, 41.00] | 33.50 [29.60, 39.00] | 0.010 |
| vent_hemoglobin_min(g/dL, median [IQR]) | 10.10 [8.70, 11.72] | 9.70 [8.10, 11.30] | 0.005 |
| vent_hemoglobin_max(g/dL, median [IQR]) | 11.70 [10.10, 13.40] | 11.00 [9.50, 12.90] | 0.004 |
| vent_lactate_min(mmol/L, median [IQR]) | 1.40 [1.00, 2.00] | 1.80 [1.20, 2.90] | <0.001 |
| vent_lactate_max(mmol/L, median [IQR]) | 2.20 [1.40, 4.18] | 3.40 [1.80, 7.10] | <0.001 |
| vent_platelet_min(10^9/L, median [IQR]) | 180.00 [117.00, 247.00] | 143.00 [75.00, 224.00] | <0.001 |
| vent_platelet_max(10^9/L, median [IQR]) | 218.00 [158.00, 295.00] | 188.00 [114.00, 279.00] | <0.001 |
| vent_potassium_min(mmol/L, median [IQR]) | 3.70 [3.30, 4.00] | 3.70 [3.30, 4.10] | 0.525 |
| vent_potassium_max(mmol/L, median [IQR]) | 4.30 [3.95, 4.90] | 4.50 [4.00, 5.10] | 0.042 |
| vent_inr_min (median [IQR]) | 1.20 [1.10, 1.44] | 1.30 [1.10, 1.70] | 0.002 |
| vent_inr_max (median [IQR]) | 1.40 [1.20, 1.70] | 1.60 [1.20, 2.25] | 0.001 |
| vent_pt_min (sec, median [IQR]) | 14.00 [12.90, 15.60] | 14.60 [13.20, 18.02] | 0.002 |
| vent_pt_max (sec, median [IQR]) | 15.00 [13.60, 17.50] | 16.95 [13.90, 21.35] | <0.001 |
| vent_sodium_min (mmol/L, median [IQR]) | 138.00 [135.00, 140.00] | 137.00 [134.00, 141.00] | 0.64 |
| vent_sodium_max (mmol/L, median [IQR]) | 141.00 [138.00, 144.00] | 141.00 [137.00, 144.00] | 0.887 |
| vent_bun_min (mg/dL, median [IQR]) | 18.00 [12.00, 30.00] | 29.00 [17.00, 45.00] | <0.001 |
| vent_bun_max (mg/dL, median [IQR]) | 23.00 [15.00, 37.00] | 34.00 [21.00, 55.00] | <0.001 |
| vent_wbc_min (10^9/L, median [IQR]) | 10.30 [6.60, 14.75] | 11.30 [6.80, 16.10] | 0.052 |
| vent_wbc_max (10^9/L, median [IQR]) | 13.80 [9.70, 19.20] | 15.30 [10.22, 20.70] | 0.091 |
| vent_ca_t_min (mmol/L, median [IQR]) | 7.80 [7.10, 8.30] | 7.60 [7.10, 8.20] | 0.068 |
| vent_ca_t_max (mmol/L, median [IQR]) | 8.30 [7.80, 8.80] | 8.20 [7.70, 8.80] | 0.614 |
| vent_rbc_min (10^12/L, median [IQR]) | 3.42 [2.93, 3.92] | 3.32 [2.76, 3.84] | 0.006 |
| vent_rbc_max (10^12/L, median [IQR]) | 3.83 [3.40, 4.44] | 3.70 [3.12, 4.28] | 0.002 |
| vent_mch_min(pg, median [IQR]) | 29.90 [28.40, 31.10] | 30.15 [28.50, 31.60] | 0.129 |
| vent_mch_max(pg, median [IQR]) | 30.60 [29.00, 31.80] | 30.70 [29.00, 32.18] | 0.116 |
| vent_mchc_min(%, median [IQR]) | 33.00 [32.00, 33.80] | 32.70 [31.70, 33.70] | 0.076 |
| vent_mchc_max(%, median [IQR]) | 33.80 [32.58, 34.90] | 33.50 [32.20, 34.80] | 0.191 |
| vent_mcv_min(fL, median [IQR]) | 89.00 [85.00, 93.53] | 90.00 [86.00, 95.00] | 0.029 |
| vent_mcv_max(fL, median [IQR]) | 91.00 [87.00, 95.00] | 92.50 [88.00, 97.00] | 0.004 |
| vent_rdw_min(%, median [IQR]) | 14.40 [13.50, 15.70] | 15.25 [14.00, 17.28] | <0.001 |
| vent_rdw_max(%, median [IQR]) | 14.80 [13.80, 16.00] | 15.50 [14.30, 17.88] | <0.001 |
| vent_albumin_min, g/dL (median [IQR]) | 2.60 [2.20, 3.10] | 2.50 [1.90, 2.90] | 0.003 |
| vent_albumin_max, g/dL (median [IQR]) | 2.80 [2.30, 3.30] | 2.80 [2.15, 3.20] | 0.112 |
| vent_bilirubin_min, g/dL (median [IQR]) | 0.65 [0.40, 1.00] | 0.80 [0.40, 2.00] | 0.003 |
| vent_bilirubin_max, g/dL (median [IQR]) | 0.70 [0.50, 1.28] | 0.90 [0.50, 3.10] | 0.001 |
| vent_ALT_min, IU/L (median [IQR]) | 32.0 [19.0, 58.8] | 34.0 [19.2, 88.8] | 0.223 |
| vent_ALT_max, IU/L (median [IQR]) | 36.0 [22.0, 73.0] | 41.0 [22.0, 128] | 0.076 |
| vent_AST_min, IU/L (median [IQR]) | 44.0 [25.0, 84.0] | 62.0 [27.2, 145] | 0.003 |
| vent_AST_max, IU/L (median [IQR]) | 52.0 [27.5, 118] | 75.5 [33.2, 232] | 0.001 |
| vent_ALP_min, IU/L (median [IQR]) | 77.0 [55.0, 104] | 88.0 [65.0, 130] | 0.001 |
| vent_ALP_max, IU/L (median [IQR]) | 82.0 [61.0, 113] | 99.0 [71.0, 141] | <0.001 |
| vent_urineoutput(mL, median [IQR]) | 1655.00 [930.00, 2504.00] | 1050.00 [549.00, 1880.00] | <0.001 |

*sysbp* systolic blood pressure, *diasbp* diastolic blood pressure, *meanbp* mean blood pressure, *resprate* respiratary rate, *tempc* temperature, *bun* blood urea nitrogen, *wbc* white blood cell, *INR* international normalized ratio, *Spo2* oxyhemoglobin saturation，*pt* prothrombin time，*rbc* red blood cell,*mch* mean hemoglobin content of red blood cells, *mchc* mean corpuscular hemoglobin concentration, *mcv* mean corpuscular volume, *rdw* red blood corpuscular volume distribution width,*aado2* alveolar-arterial oxygen gradient ,*po2* oxygen partial pressure, *pco2* partial pressure of carbon dioxide, *ca_t* calcium, *peak_insp_pressure* peak inspiratory pressure, *resp_rate_set* setting respiratory rate , *ALT* Alanine Aminotransferase, *AST* Asparate Aminotransferase, *ALP* Alkaline Phosphatase, *vent* ventilation, *max* maximum, *min* minimum

Note: Variable name with the prefix of vent means the data was collected within the first 24 hours of invasive ventilation.

**TableS3. Characteristics of the internal validation cohort comparing survived vs non-survived patients.**

|  | Survivor  (n=225) | Non-Survivor  (n=98) | *P* |
| --- | --- | --- | --- |
| Age, yr (median [IQR]) | 58.00 [46.39, 69.80] | 64.75 [54.72, 78.92] | <0.001 |
| Respiratory rate_mean, breaths/min (mean±SD) | 21.58 [18.64, 25.97] | 22.89 [20.09, 25.92] | 0.058 |
| INR_max (median [IQR]) | 1.40 [1.20, 1.70] | 1.40 [1.16, 1.70] | 0.941 |
| RDW_min, % (median [IQR]) | 14.30 [13.40, 16.00] | 15.15 [14.00, 17.02] | 0.001 |
| AaDO_2__max, mmHg (median [IQR]) | 412.45 [228.65, 575.25] | 549.00 [326.44, 598.74] | <0.001 |
| Albumin_min, g/dL (mean ± SD) | 2.74 ±0.64 | 2.46±0.70 | 0.005 |
| Albumin_max, g/dL (mean ± SD) | 2.88 ±0.64 | 2.68±0.73 | 0.053 |
| Bilirubin_min, g/dL (median [IQR]) | 0.50 [0.30, 1.17] | 0.75 [0.50, 1.80] | 0.013 |
| Bilirubin_max, g/dL (median [IQR]) | 0.60 [0.40, 1.30] | 0.80 [0.50, 1.95] | 0.013 |
| ALT_min, IU/L (median [IQR]) | 32.0 [19.8, 58.5] | 30.5 [21.0, 69.5] | 0.595 |
| ALT_max, IU/L (median [IQR]) | 33.5 [20.0, 63.2] | 35.0 [23.2, 83.0] | 0.256 |
| AST_min, IU/L (median [IQR]) | 39.0 [25.0, 89.8] | 44.0 [24.0, 124] | 0.392 |
| AST_max, IU/L (median [IQR]) | 43.5 [26.0, 96.0] | 51.0 [31.0, 175] | 0.164 |
| ALP_min, IU/L (median [IQR]) | 84.5 [64.0, 114] | 90.0 [62.0, 128] | 0.501 |
| ALP_max, IU/L (median [IQR]) | 90.0 [65.2, 126] | 97.0 [63.0, 159] | 0.304 |
| vent_Tempc_mean, ℃ (median [IQR]) | 37.17 [36.77, 37.68] | 36.85 [36.35, 37.38] | <0.001 |
| vent_Lactate_max, mmol/L (median [IQR]) | 2.20 [1.30, 4.50] | 2.50 [1.55, 5.15] | 0.048 |
| vent_BUN_min, mg/dL (median [IQR]) | 18.00 [13.00, 29.00] | 27.00 [18.75, 42.50] | <0.001 |
| vent_WBC_min, 10^9/L (median [IQR]) | 10.40 [7.70, 15.20] | 11.85 [8.03, 16.73] | 0.190 |
| vent_Albumin_min, g/dL (mean ± SD) | 2.67 ±0.62 | 2.43 ±0.74 | 0.027 |
| vent_Albumin_max, g/dL (mean ± SD) | 2.80 ±0.63 | 2.62 ±0.79 | 0.114 |
| vent_Bilirubin_min, g/dL (median [IQR]) | 0.60 [0.30, 1.20] | 0.80 [0.50, 1.80] | 0.013 |
| vent_Bilirubin_max, g/dL (median [IQR]) | 0.60 [0.40, 1.30] | 1.00 [0.50, 2.10] | 0.008 |
| vent_ALT_min, IU/L (median [IQR]) | 32.0 [19.0, 66.0] | 32.0 [21.0, 68.0] | 0.463 |
| vent_ALT_max, IU/L (median [IQR]) | 34.0 [19.8, 67.5] | 37.0 [23.0, 86.0] | 0.279 |
| vent_AST_min, IU/L (median [IQR]) | 38.5 [23.0, 91.8] | 51.5 [26.2, 132] | 0.134 |
| vent_AST_max, IU/L (median [IQR]) | 44.0 [25.0, 96.0] | 55.5 [27.5, 192] | 0.115 |
| vent_ALP_min, IU/L (median [IQR]) | 80.0 [60.5, 110] | 87.5 [61.2, 131] | 0.3 |
| vent_ALP_max, IU/L (median [IQR]) | 83.0 [64.0, 122] | 95.5 [62.2, 150] | 0.23 |
| Subgroup of ARDS, n (%) | | | 0.123 |
| Direct (pulmonary) ARDS | 139 (61.8) | 70 (71.4) |  |
| Indirect (extrapulmonary) ARDS | 86 (38.2) | 28 (28.6) |  |
| Severity score | | | |
| SAPS II (median [IQR]) | 39.00 [32.00, 51.50] | 55.00 [44.50, 66.50] | <0.001 |
| OASIS (mean (SD)) | 35.96 (8.38) | 39.70 (9.81) | 0.001 |
| SOFA (median [IQR]) | 6.00 [4.00, 9.00] | 8.00 [5.00, 12.00] | 0.001 |
| APACHE IV (median [IQR]) | 71.50 [56.00, 91.75] | 88.00 [67.50, 110.50] | 0.003 |
| APPS (median [IQR]) | 5.00 [5.00, 7.00] | 6.00 [5.00, 7.00] | 0.002 |

*INR* international normalized ratio, *RDW* red blood cell distribution width, *AaDO_2_* alveolo-arterial oxygen difference*, Tempc* temperature, *BUN* blood urea nitrogen, *WBC* white blood cell, *ALT* Alanine Aminotransferase, *AST* Asparate Aminotransferase, *ALP* Alkaline Phosphatase, *SAPS II* simplified acute physiology score II, *SOFA* sequential organ failure assessment, *OASIS* oxford acute severity of illness score, *APACHE IV* acute physiology and chronic health evaluation IV, *APPS* Age, PaO_2_/FiO_2_, and Plateau Pressure Score, *vent* ventilation, *max* maximum, *min* minimum

Note: Variable name with the prefix of *vent* means the data was collected within the first 24 hours of invasive ventilation.

**Table S4. Characteristics of the external validation cohort comparing survived vs non-survived patients.**

|  | Survivor  (n=355) | Non-Survivor  (n=166) | *P* |
| --- | --- | --- | --- |
| Age, yr (median [IQR]) | 60.00 [47.00, 70.00] | 64.00 [54.25, 75.00] | 0.001 |
| Gender (male) | 217 (61.1%) | 101 (60.8%) | >0.999 |
| Respiratory rate_mean, breaths/min (mean ± SD) | 22.45±4.38 | 22.35±4.32 | 0.806 |
| INR_max (median [IQR]) | 1.40 [1.20, 1.80] | 1.60 [1.30, 2.48] | <0.001 |
| RDW_min, % (median [IQR]) | 14.70 [13.55, 16.00] | 15.35 [14.00, 17.50] | 0.001 |
| AaDO_2__max, mmHg (median [IQR]) | 395.25 [243.62, 561.00] | 438.45 [270.26, 578.56] | 0.055 |
| Albumin_min, g/dL (median [IQR]) | 2.80 [2.40, 3.20] | 2.70 [2.20, 3.30] | 0.464 |
| Albumin_max, g/dL (median [IQR]) | 2.90 [2.50, 3.30] | 2.85 [2.40, 3.50] | 0.821 |
| Bilirubin_min, g/dL (median [IQR]) | 0.80 [0.40, 1.60] | 1.00 [0.50, 3.65] | 0.001 |
| Bilirubin_max, g/dL (median [IQR]) | 0.90 [0.50, 2.30] | 1.30 [0.60, 4.98] | 0.001 |
| ALT_min, IU/L (median [IQR]) | 34.0 [19.0, 72.0] | 37.0 [23.0, 108] | 0.299 |
| ALT_max, IU/L (median [IQR]) | 42.5 [21.0, 121] | 44.0 [26.0, 194] | 0.175 |
| AST_min, IU/L (median [IQR]) | 58.0 [29.2, 127] | 69.5 [37.8, 230] | 0.02 |
| AST_max, IU/L (median [IQR]) | 72.0 [34.0, 204] | 110 [44.8, 447] | 0.012 |
| ALP_min, IU/L (median [IQR]) | 72.0 [55.0, 102] | 90.0 [64.2, 133] | <0.001 |
| ALP_max, IU/L (median [IQR]) | 82.5 [60.8, 115] | 104 [74.0, 156] | <0.001 |
| vent_Tempc_mean, ℃ (median [IQR]) | 37.22 [36.87, 37.62] | 36.96 [36.63, 37.49] | <0.001 |
| vent_Lactate_max, mmol/L (median [IQR]) | 2.40 [1.60, 4.45] | 3.45 [2.20, 6.60] | <0.001 |
| vent_BUN_min, mg/dL (median [IQR]) | 22.00 [13.00, 37.00] | 29.00 [18.00, 44.00] | <0.001 |
| vent_WBC_min, 10^9/L (median [IQR]) | 11.30 [7.30, 16.05] | 11.55 [7.10, 18.10] | 0.505 |
| vent_Albumin_min, g/dL (median [IQR]) | 2.70 [2.30, 3.20] | 2.70 [2.20, 3.20] | 0.471 |
| vent_Albumin_max, g/dL (median [IQR]) | 2.90 [2.50, 3.30] | 2.80 [2.40, 3.40] | 0.938 |
| vent_Bilirubin_min, g/dL (median [IQR]) | 0.70 [0.40, 1.70] | 1.00 [0.55, 3.55] | <0.001 |
| vent_Bilirubin_max, g/dL (median [IQR]) | 0.90 [0.50, 2.30] | 1.30 [0.60, 5.30] | 0.001 |
| vent_ALT_min, IU/L (median [IQR]) | 33.0 [19.0, 75.0] | 37.0 [23.0, 112] | 0.215 |
| vent_ALT_max, IU/L (median [IQR]) | 43.0 [22.0, 120] | 47.0 [26.0, 183] | 0.142 |
| vent_AST_min, IU/L (median [IQR]) | 58.0 [29.0, 128] | 70.5 [38.0, 215] | 0.017 |
| vent_AST_max, IU/L (median [IQR]) | 74.0 [33.5, 206] | 109 [45.2, 442] | 0.011 |
| vent_ALP_min, IU/L (median [IQR]) | 72.0 [55.0, 102] | 89.0 [63.5, 131] | 0.001 |
| vent_ALP_max, IU/L (median [IQR]) | 81.0 [61.0, 113] | 102 [71.5, 154] | <0.001 |
| Subgroup of ARDS, n (%) | | | 0.365 |
| Direct (pulmonary) ARDS | 230 (64.8) | 100 (60.2) |  |
| Indirect (extrapulmonary) ARDS | 86 (38.2) | 28 (28.6) |  |
| Severity score | | | |
| SAPS II (median [IQR]) | 46.00 [37.00, 57.00] | 55.00 [47.00, 67.00] | <0.001 |
| SOFA (median [IQR]) | 9.00 [6.00, 11.00] | 11.00 [8.00, 14.00] | <0.001 |
| OASIS (median [IQR]) | 42.00 [37.00, 48.00] | 48.00 [42.00, 53.75] | <0.001 |
| APPS (median [IQR]) | 5.00 [5.00, 6.00] | 6.00 [5.00, 7.00] | <0.001 |

*INR* international normalized ratio, *RDW* red blood cell distribution width, *AaDO_2_* alveolo-arterial oxygen difference*, Tempc* temperature, *BUN* blood urea nitrogen, *WBC* white blood cell, *ALT* Alanine Aminotransferase, *AST* Asparate Aminotransferase, *ALP* Alkaline Phosphatase, *SAPS II* simplified acute physiology score II, *OASIS* oxford acute severity of illness score, *APPS* Age, PaO_2_/FiO_2_, and Plateau Pressure Score, *vent* ventilation, *max* maximum, *min* minimum

Note: Variable name with the prefix of vent means the data was collected within the first 24 hours of invasive ventilation.


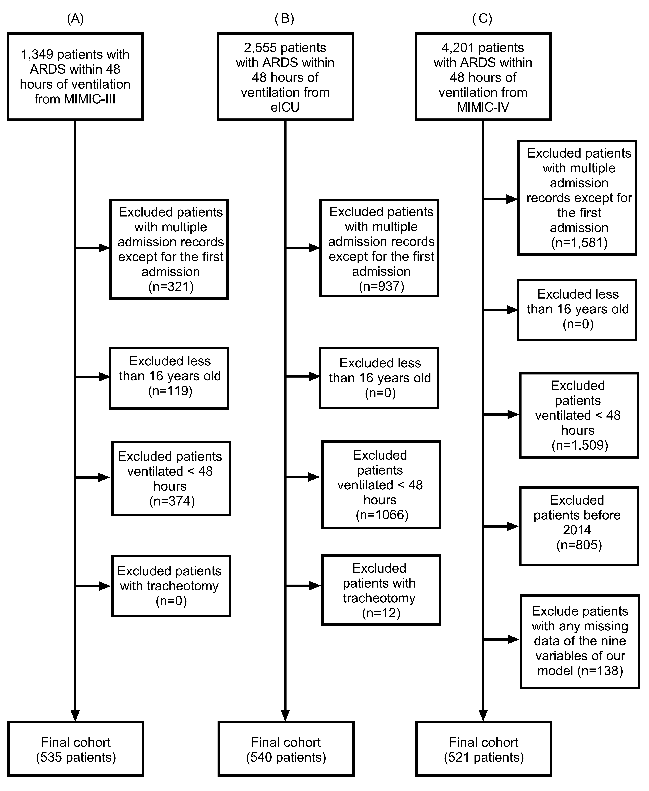


Figure S1. The detailed process of data extraction. (A) MIMIC-III; (B)eICU; (C) MIMIC-IV.

# Patients in external validation cohort with any missing data ascertained at ICU admission including age, mean of respiratory rate, the maximum of INR and alveolo-arterial oxygen difference, and the minimum of RDW, and data collected at the beginning of invasive ventilation including mean of temperature, the maximum of lactate, the minimum of blood urea nitrogen and white blood cell counts were excluded.

(A) LASSO coefficient profiles of the 176 features.


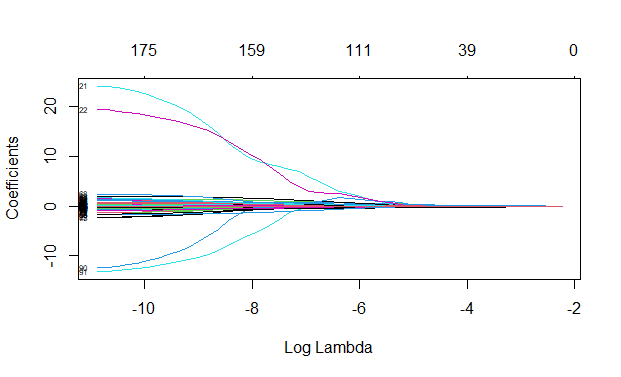


(B) Tuning parameter (λ) selection in the LASSO model used 10-fold cross-validation via minimum criteria.


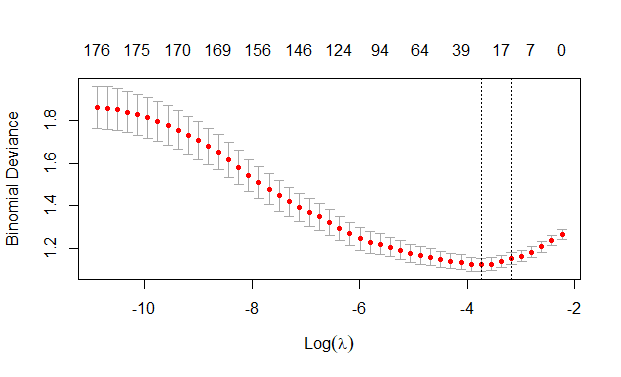


Figure S2. Feature selection using the least absolute shrinkage and selection operator (LASSO) binary logistic regression model. (A) LASSO coefficient profiles of the 176 features. (B) Tuning parameter (λ) selection in the LASSO model used 10-fold cross-validation via minimum criteria


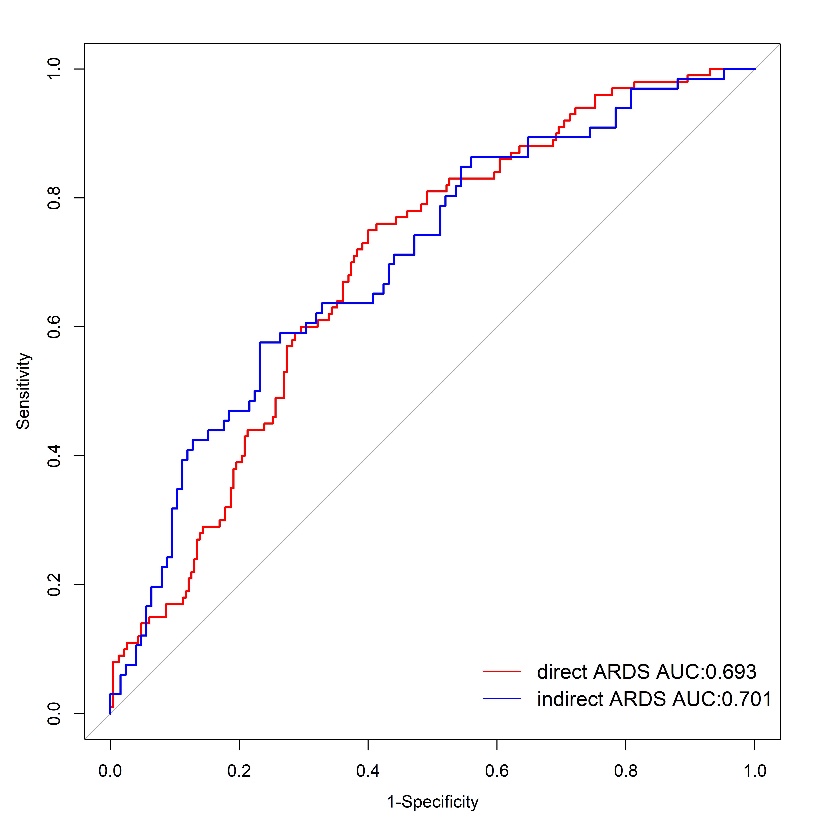


Figure S3. The ROC curves of our model validated in cohort of direct ARDS and indirect ARDS.

De Long method, direct ARDS vs. indirect ARDS *P*=0.858


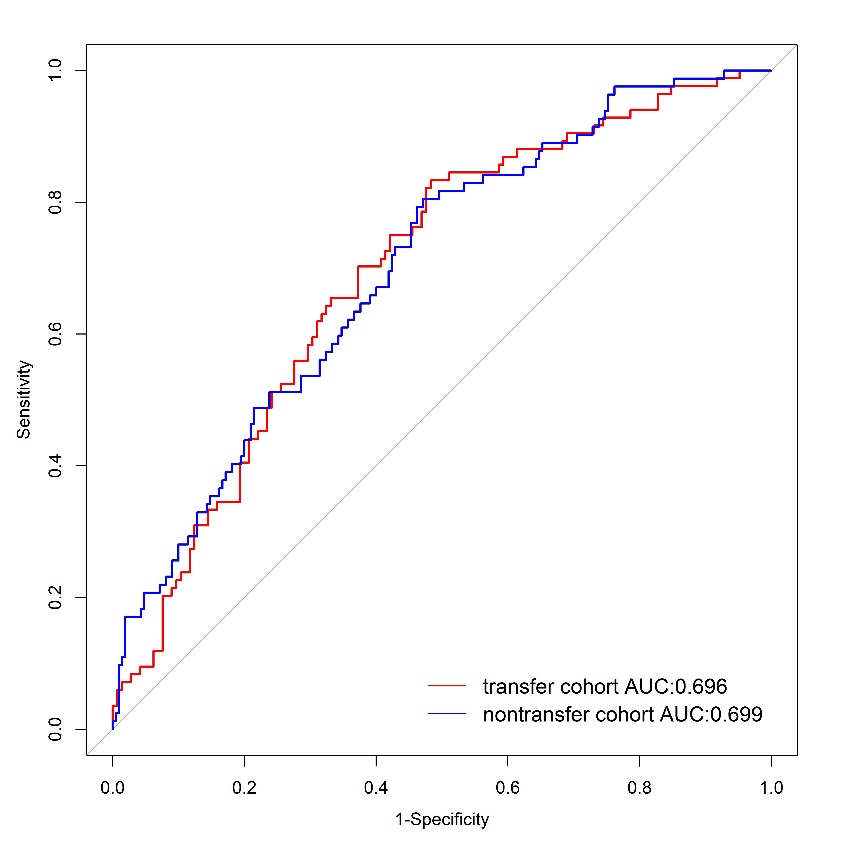


Figure S4. The ROC curves of our model validated in cohort of transferred and non-transferred.

De Long method, transferred vs. non-transferred *P*=0.858
